# Optimizing planting density and nitrogen application to enhance profit and nitrogen use of summer maize in Huanghuaihai region of China

**DOI:** 10.1038/s41598-022-06059-0

**Published:** 2022-02-17

**Authors:** HaiYan Zhang, ChengRan Zhang, Peng Sun, XuWen Jiang, GuangHai Xu, JinZhong Yang

**Affiliations:** Qingdao Agricultural University, Qingdao, China

**Keywords:** Plant sciences, Plant physiology

## Abstract

Low planting density and irrational nitrogen (N) fertilization are two common practices in conventional cropping of smallholder maize production in Huanghuaihai region of China. A 2-year field experiment was carried out to study the effects of N application and planting density on maize phenology, dry matter accumulation, profit, yield, N uptake and efficiency indices. The experiments included three N application levels (120 kg ha^−1^, N1; 180 kg ha^−1^, N2; 240 kg ha^−1^, N3) and three planting densities (60,000 plants ha^−1^, D1; 75,000 plants ha^−1^, D2; 90,000 plants ha^−1^, D3). Increasing N input and planting density delayed the physiological maturity and enhanced dry matter accumulation. Comparing with the traditional N3 level, grain yield and profit were kept stable at N2 level and decreased at N1 level, partial factor productivity of applied N (PFP_N_) and nitrogen efficiency ratio (NER) were increased with the decreasing of N level. Comparing with the traditional D1 density, grain yield, profit and PFP_N_ were increased at D2 density and then kept stable at D3 density, NER was kept stable at D2 density and then decreased at D3 density. Based on the predicted maximum profit, the optimal combinations of N application and planting density were 199 kg ha^−1^ and 81,081 plants ha^−1^ in 2017, and 205 kg ha^−1^ and 84,782 plants ha^−1^ in 2018. The two optimal combinations had an increase of 17.6% for grain yield, 39.8% for PEP_N_, 3.6% for NRE than the traditional N3D1 treatment. Therefore, an appropriate combination of increased planting density with reduced N application could enhance profit and nitrogen use of summer maize in Huanghuaihai region of China.

## Introduction

The 2020 World Population Data Sheet indicates that world population is expected to increase from 7.8 billion in 2020 to 9.9 billion by 2050. This is likely to drastically increase food demand over the coming decades. As a staple crop in the world, maize cultivation is essential for yield improvement to meet the increasing food demand.

An evaluation of long-term studies has shown that 40 to 60% of crop yield can be attributed to fertilizer inputs^1,2^. Nitrogen (N) fertilizer is one of the most vital inputs in maize production^3^. Increasing N application rate is an effective way of obtaining high yield^4,5^. However, excessive fertilization often leads to not only low N use efficiency but also serious threats to environment and human health^3,6^. Besides N fertilizer, increasing planting density is a way for increasing grain yield, as it improved the ability of the crop canopy to capture water, nutrients and light^7,8^. However, crowded maize plants under high planting density, will result in resource competition between plants and consequently lead to a yield reduction per plant^9^. Therefore, optimal N input and planting density are critical for maize production.

Optimal planting density for high yield should be associated with an appropriate N application^10,11^. In super high-yield maize experiments, an appropriate increase of planting density and a reasonable reduction of N application can not only achieve high maize yield but also raise the N utilization efficiency in Northwest China^12^. In China, Huanghuaihai region is one major maize planting area, with planting area and the production occupying 34.7% and 36.8% of the whole China^13^, respectively. In this region, some problems exist with the traditional management practices of smallholder farming, such as low planting density and irrational N fertilizer^14,15^. Therefore, agricultural strategy that aims to obtain high profit and resource efficiency by means of optimizing planting density and N application rate is on demand. In this study, we hypothesized that, in Huanghuaihai region of China, high yield, high N use efficiency, and high economic return could be obtained by increasing planting density and optimizing N application rate. Therefore, the objectives of this study were to (1) determine the impacts of N application rate and planting density on maize phenology, dry matter, yield, profit, and N use; and (2) to determine the combination of planting density and N application rate which maximize the profit and N use based on smallholder farming in Huanghuaihai region in China.

## Results

### Days to tasseling, silking and physiological maturity

As shown in Table 1, planting density had significant effects on days to tasselling and silking in 2017, and both N rate and planting density had significant effects on days to tasselling, silking and physiological maturity in 2018 (Table 1). The decreasing N rate delayed the days to tasselling and silking, while shortened the days to physiological maturity especially in 2018. The significantly shortest period to tasseling (47.5 days) and silking (50.0 days) while longest period to physiological maturity (102.7 days) under N3 treatment were recorded according to 2 years’ data. With increasing planting density, days to tasseling, silking and physiological maturity were delayed. The significantly longest period to tasseling (48.4 days), silking (51.2 days) and physiological maturity (102.9 days) under D3 treatment were recorded over two years.

**Table 1:** Phenology in 2017 and 2018.

N rate	Planting density	Tasselling (days)		Silking (days)		Physiological maturity (days)	
		2017	2018	2017	2018	2017	2018
N1	D1	47.0b	48.0cd	49.0c	50.0de	100.7d	101.3d
	D2	47.0b	49.0b	49.0c	51.3bc	101.3bcd	102.7bc
	D3	48.7a	49.7a	51.0a	52.7a	101.0cd	102.7bcd
N2	D1	47.0b	47.7d	49.0c	49.7ef	101.7abcd	101.3d
	D2	47.3b	48.3c	49.7bc	50.3de	101.7abcd	102.3cd
	D3	47.7b	49.0b	50.3ab	51.7b	102.7a	104.0ab
N3	D1	47.0b	47.0e	49.0c	49.0f	101.7abcd	102.0cd
	D2	47.3b	48.0cd	49.7bc	50.7cd	102.0abc	103.3bc
	D3	47.3b	48.3c	50.0b	51.3bc	102.3ab	105.0a
Significance	N rate (N)	ns	**	ns	***	ns	*
	Density (D)	*	***	***	***	ns	***

The three N rates are 120 (N1), 180 (N2), and 240 (N3) kg ha^−1^. The three planting density levels are 60,000 (D1), 75,000 (D2), and 90,000 (D3) plants ha^−1^.

*ns* no significant difference.

Means within a column followed by the different letter are not significantly different at *P* < 0.05 as determined by the LSD test.

*, **, and *** indicate significant difference at 0.05, 0.01 and 0.001 level, respectively.

### Dry matter accumulation

**Figure 1:**
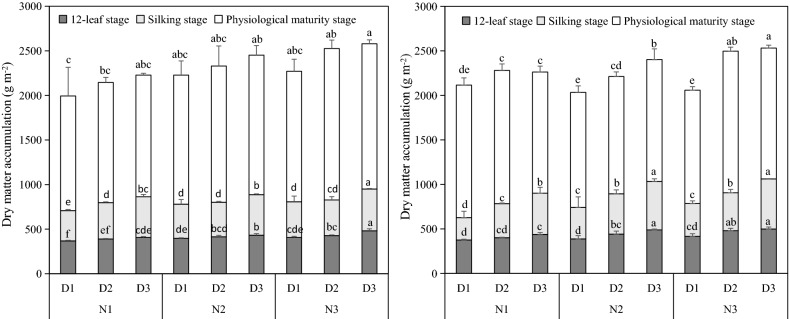
Dry matter accumulation of 12 leaf stage, silking stage and maturity stage in 2017 (left) and 2018 (right). The three N rates are 120 (N1), 180 (N2), and 240 (N3) kg ha^−1^. The three planting density levels are 60,000 (D1), 75,000 (D2), and 90,000 plants ha^−1^ (D3). Symbols represent means ± standard error. Vertical bars indicate standard error. Different letters within the same stage are significantly different at the 0.05 probability level.

With the increase of either N rate or planting density, dry matter accumulation at 12-leaf stage, silking stage and physiological maturity stage were increased (Fig. 1). Compared to traditional N3 treatment, dry matter accumulation under N1 treatment showed a 11.6%, 12.2%, and 9.4% decrease in these three stages over 2 years. Compared to traditional D1 treatment, dry matter accumulation under D3 treatment showed a 16.8%, 28.4%, and 13.8% increase in these three stages over 2 years. The maximal dry matter accumulation was obtained with N3 × D3 treatment.

### Maize yield and profit

N rate had significant effects on 1000-grain weight, grain yield and profit and didn’t have significant effects on ear number and grains per ear, and planting density had significant effects on all these five indices (Table 2). Increasing N rate caused 1000-grain weight, grain yield and profit increases with the peak showing at N2 treatment and remained stable at traditional N3 treatment. After averaging the effects of planting density, compared to N1 treatment, traditional N3 treatment increased 1000-grain weight, grain yield and profit by 2.6%, 5.3% and 2.7%, respectively. Increasing planting density resulted in an increased ear number, a decreased grains per ear and 1000-grain weight, an increased initially and then stable yield and profit. After averaging the effects of N rate, ear number, grains per ear, 1000-grain weight, grain yield and profit of D3 treatment were 39.2%, − 8.7%, − 7.2%, 16.6% and 16.2% higher than the traditional D1 treatment, respectively. According to the 2 years’ results, the greatest grain yield and profit was easily obtained for the combination of N2 or N3 and D2 or D3.

**Table 2:** Maize ear number, grains per ear, 1000-grain weight, grain yield and profit in 2017 and 2018.

N rate	Planting density	Ear number (1000 ears ha^−1^)		Grain number per ear		Grain weight (moisture 14%, g)		Grain yield (moisture 14%, t ha^−1^)		Profit ($ ha^−1^)	
		2017	2018	2017	2018	2017	2018	2017	2018	2017	2018
N1	D1	59.9e	54.5e	546bc	516a	327.4b	322.0a	9.87d	9.08d	2488e	2274c
	D2	73.5cd	66.3c	507d	509b	314.6c	314.7b	11.20b	10.40b	2823cd	2607b
	D3	78.7abc	77.7a	479e	490d	307.5c	294.2d	11.23b	10.96a	2809d	2734a
N2	D1	58.9e	56.3de	550b	516a	338.5a	324.3a	10.30c	9.40cd	2564e	2321c
	D2	76.1bcd	65.6c	510d	517a	323.8b	323.0a	11.99a	10.91a	2997a	2705a
	D3	80.2ab	79.5a	495de	487d	313.8c	305.2c	11.78a	11.15a	2916ab	2748a
N3	D1	56.5e	56.8d	573a	518a	337.1a	327.2a	10.35c	9.56c	2537e	2326c
	D2	71.7d	68.4b	530c	509b	327.3b	323.8a	11.93a	11.17a	2939ab	2735a
	D3	82.1a	79.1a	487e	498c	308.6c	305.3c	11.87a	11.23a	2901bc	2729a
Significance	N rate (N)	ns	ns	ns	ns	*	*	**	*	*	ns
	Density (D)	***	***	***	***	***	***	***	***	***	***

*ns* no significant difference.

Means within a column followed by the different letters are significantly different at *P* < 0.05. N is N rate, and D is planting density. The three N rates are 120 (N1), 180 (N2), and 240 (N3) kg ha^−1^. The three planting density levels are 60,000 (D1), 75,000 (D2), and 90,000 plants ha^−1^ (D3).

*Significance at the *P* < 0.05 level.

**Significance at the *P* < 0.01 level.

***Significance at the *P* < 0.001 level.

### N uptake and N efficiency indices

Significant effects were observed from N rate and planting density on total N uptake, PFP_N_ and NER. As N rate increased, the total N uptake was increased, while PFP_N_ and NER were decreased. Traditional N3 treatment led to the maximal total N uptake and the minimal in PFP_N_ and NER at all planting densities. Planting density had a different effect on these three indices. With the increased planting density, the total N uptake was increased, while PFP_N_ was increased at D2 treatment and then kept stable at D3 treatment, NRE was kept stable at D2 treatment and then decreased at D3 treatment. Based on the 2 years’ results, the high PFP_N_ and NER were obtained at the combination of low N application and suitable planting density.

### Optimizing N application rate and planting density

**Figure 2:**
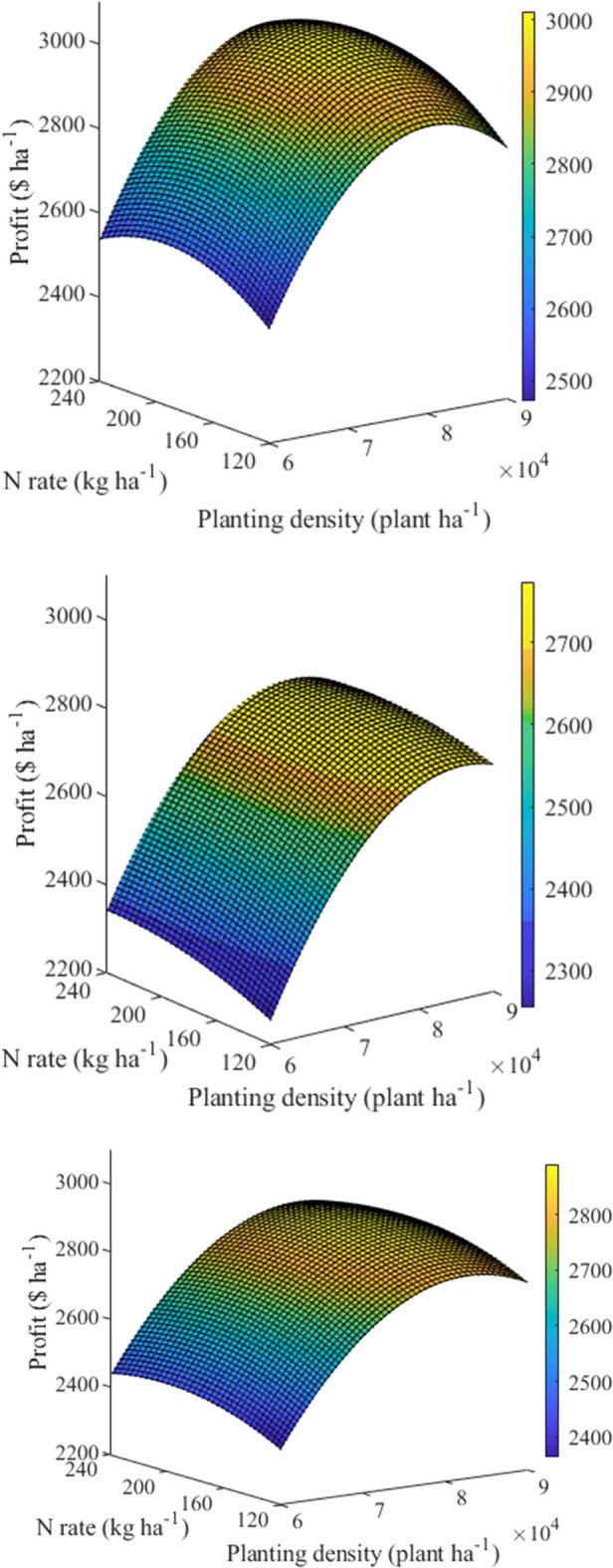
Quadratic polynomial trend surface fitting of profit, N application rate, and planting density. *x* was set as N application rate, *y* was set as planting density, and *z* was set as profit to show the effects of N application rate and planting density on the profits of 2017 (up) and 2018 (middle) and 2 years’ mean (down).

To determine the optimal N application rate and planting density for profit, the relationships between N application and planting density and the profit was analysed (Fig. 2). The response surfaces that demonstrating the combined effect of N application and planting density on the profit were convex. Namely, with the increased N application and planting density, the profit was firstly increased, then reached a peak value, and finally decreased.

According to the second-order polynomials in Table 3, the predicted maximum profit was 3012 $ ha^−1^ (199 kg ha^−1^ and 81,081 plants ha^−1^) in 2017 and 2774 $ ha^−1^ (205 kg ha^−1^ and 84,782 plants ha^−1^) in 2018. Based on the above combination of N rate and planting density, the grain yield (Max_Profit_ GY), PFP_N_ (Max_Profit_ PFP_N_) and NER (Max_Profit_ NER) were 12.1 t ha^−1^, 60.9 kg kg^−1^ and 41.1% in 2017, 11.3 t ha^−1^, 54.7 kg kg^−1^ and 38.6% in 2018. Compared to the traditional N3D1 treatment, Max_Profit_ GY, Max_Profit_ PFP_N_ and Max_Profit_ NER were increased by 17.6%, 39.8% and 3.6% in these 2 years, respectively.

**Table 3:** The quadratic polynomial trend surface equations, and yield, PFP_N_ and NER.

Year	Equation	*R*^2^	*p*	*x*	*y*	*z*	GY	PFP_N_	NER
2017	z = − 3978 + 7.421x + 0.154y + 1.205E−05xy − 0.0211x^2^ − 9.659E−07y^2^	0.992	**	199	81,081	3012	12.13	60.90	41.12
2018	z = − 2897 + 4.029x + 0.124y − 1.593E−05xy − 0.0065x^2^ − 7.121E−07y^2^	0.989	**	205	84,782	2774	11.32	54.66	38.56
Mean	z = − 3438 + 5.725x + 0.139y − 1.942E−06xy − 0.0138x^2^ − 8.390E−07y^2^	0.992	**	201	82,680	2890	11.71	57.97	39.92

*R*^2^ the coefficient of determination, *x* represents the N application rate (kg ha^−1^), *y* represents the planting density (plant ha^−1^), *z* represents the profit, *GY* represents the grain yield (t ha^−1^), *PFP*_*N*_ represents partial factor productivity of applied N (kg kg^−1^), *NER* represents nitrogen efficiency ratio (%).

**Represents significance at the 0.01 probability level.

## Discussion

In this study, the plants at high planting density took longer time to tasseling, silking, and physiological maturity than the plants at low planting density. This indicated that dense planting may have slowed down plant development owing to more competition between plants^16^. A similar result was reported in earlier studies as dense population induced lengthening of the time to phenological characteristics^17–19^. In this study, increase in N input induced the advance in days to tasseling and silking. It may be due to quick growth under high nitrogen level. However, the plants took more time to physiological maturity with the increasing rate of N application. Namely, increasing N rate delayed the reproductive growth period of maize. It could be because the plants with more N application were remained green for longer period, which caused longer maturity period. The result was consistent with that of Shresth et al.^18^. The longer maturity period under high N and planting density conditions could be conducive to the increase of grain yield.

In our study, high N input or high planting density enhances the dry matter accumulation and grain yield. There was a significant positive correlation between the dry matter and the grain yield^20^. Furthermore, grain yield was extremely affected by the assimilate allocation balance between vegetative and reproductive organs^11,21,22^. The longer reproductive growth period and more photosynthates availability with high N application induced the greater assimilates allocation to the seeds during grain filling, which improved grains per ear and grain weight. Increased plant density induced leaf senescence of lower leaf and then decreased the biomass accumulation, resulting in a lower yield per plant^23^. Also, greater inter-plant competition for light, water and nutrients with excessive planting density increased ear tip-barrenness which decreased grains per ear and grain weight. Yield improvement in response to higher density might be owing to maximize light interception during the vital period for grain set^24^. At the same time, the increased ear number per ha resulted from excessive density might offset the decrease of grains per ear and grain weight. These can be the reasons for the stable yield between D3 treatment and D2 treatment. Under high planting density, a moderate decrease of leaf source through leaf removal enhanced photosynthetic performance and improved the post-silking dry matter accumulation and harvest index, and thus the grain yield^25,26^. This indicates that grain yield at high density could achieve further improvement through optimal management practices. In this study, N3D3 obtained 16.1% higher grain yield than the traditional N3D1, a great yield increase of summer maize in smallholder fields in Huanghuaihai region of China.

According to the former results^20,27,28^, the dry matter was the driving force of the enhanced N uptake. In this study, the increases both N application rate and planting density improve the dry matter accumulation, which causes a high N absorption and assimilation level. Therefore, the increased N input or planting density maintained a high N uptake. In this study, PFP_N_ and NER decreased with increased N rate. This is similar to the results of previous studies^12,15^. PFP_N_ and NER were found to have a different change with planting density, and the medium D2 density had higher PFP_N_ and NER than the low D1 density and high D3 density. This indicates that extremely high planting density is not helpful for the increase of PFP_N_ and NER. This result is consistent with that of Zhang et al.^12^ and can possibly be explained by the fact that excessive planting density results in a non-significant yield increase because of competition between plants and much more total N uptake.

Nutrient competition, mainly with respect to N, can be intensified by the increased planting density. In this study, a significant interaction between N application and planting density (Tables 2, 4) is observed. Therefore, it should be possible to optimize N application and planting density to regulate a trade-off between the three yield components (ear number, grains per ear and 1000-grain weight) to achieve high profit and N use. According to the two years’ results in this study, when the predicted maximal profit of 2890 dollar ha^−1^ was achieved, N application rate and planting density were 201 kg ha^−1^ and 82,680 plants ha^−1^, respectively. The yield, PFP_N_ and NER under this condition were higher than the traditional N3D1 treatment. Therefore, reduced N application rate and increased planting density improved not only the profit but also grain yield, PFP_N_ and NER for smallholder farming in Huanghuaihai region. Also, this study provides an important reference for determining the optimal combination of N application rate and planting density to obtain the highest profit under certain ecological conditions.

**Table 4:** The total N uptake, partial factor productivity of applied N (PFP_N_), and nitrogen efficiency ratio (NER) in 2017 and 2018.

N rate	Planting density	Total N uptake (kg ha^−1^)		PFP_N_ (kg kg^−1^)		NER (%)	
		2017	2018	2017	2017	2018	2018
N1	D1	224.6f	204.4g	82.3b	75.7c	44.0a	44.4a
	D2	258.0d	241.0e	93.3a	86.7b	43.4a	43.2ab
	D3	267.5d	262.7c	93.6a	91.3a	42.0ab	41.7bc
N2	D1	241.7e	224.1f	57.2d	52.2e	42.6a	41.9bc
	D2	281.7c	267.7c	66.6c	60.6d	42.6a	40.8c
	D3	295.1b	291.5b	65.4c	62.0d	39.9bcd	38.3d
N3	D1	262.2d	254.2d	43.1f	39.8g	39.5cd	37.6de
	D2	298.2b	290.3b	49.7e	46.5f	40.0bc	38.5d
	D3	315.9a	310.2a	49.5e	46.8f	37.6d	36.2e
Significance	N rate (N)	***	***	***	***	**	***
	Density (D)	***	***	***	***	**	***

*ns* no significant difference.

Means within a column followed by the different letters are significantly different at *P* < 0.05. N is N rate, and D is planting density. The three N rates are 120 (N1), 180 (N2), and 240 (N3) kg ha^−1^. The three planting density levels are 60,000 (D1), 75,000 (D2), and 90,000 plants ha^−1^ (D3).

**Significance at the *P* < 0.01 level.

***Significance at the *P* < 0.001 level.

## Conclusions

Maize profit and nitrogen use were affected by N application and planting density. Compared to traditional practices, the combination of 37.8% increase in planting density and 16.3% reduction in N application achieved the maximal profit. Under this optimal combination, increases of 17.6% for grain yield, 39.8% for PEP_N_, 3.6% for NRE were achieved than the traditional practices. Therefore, an appropriate increase of planting density and a reasonable reduction of N application can enhance profit, increase grain yield, reduce fertilizer input, and enhance nitrogen use of summer maize in Huanghuaihai region of China.

## Materials and methods

### Experimental site

**Figure 3:**
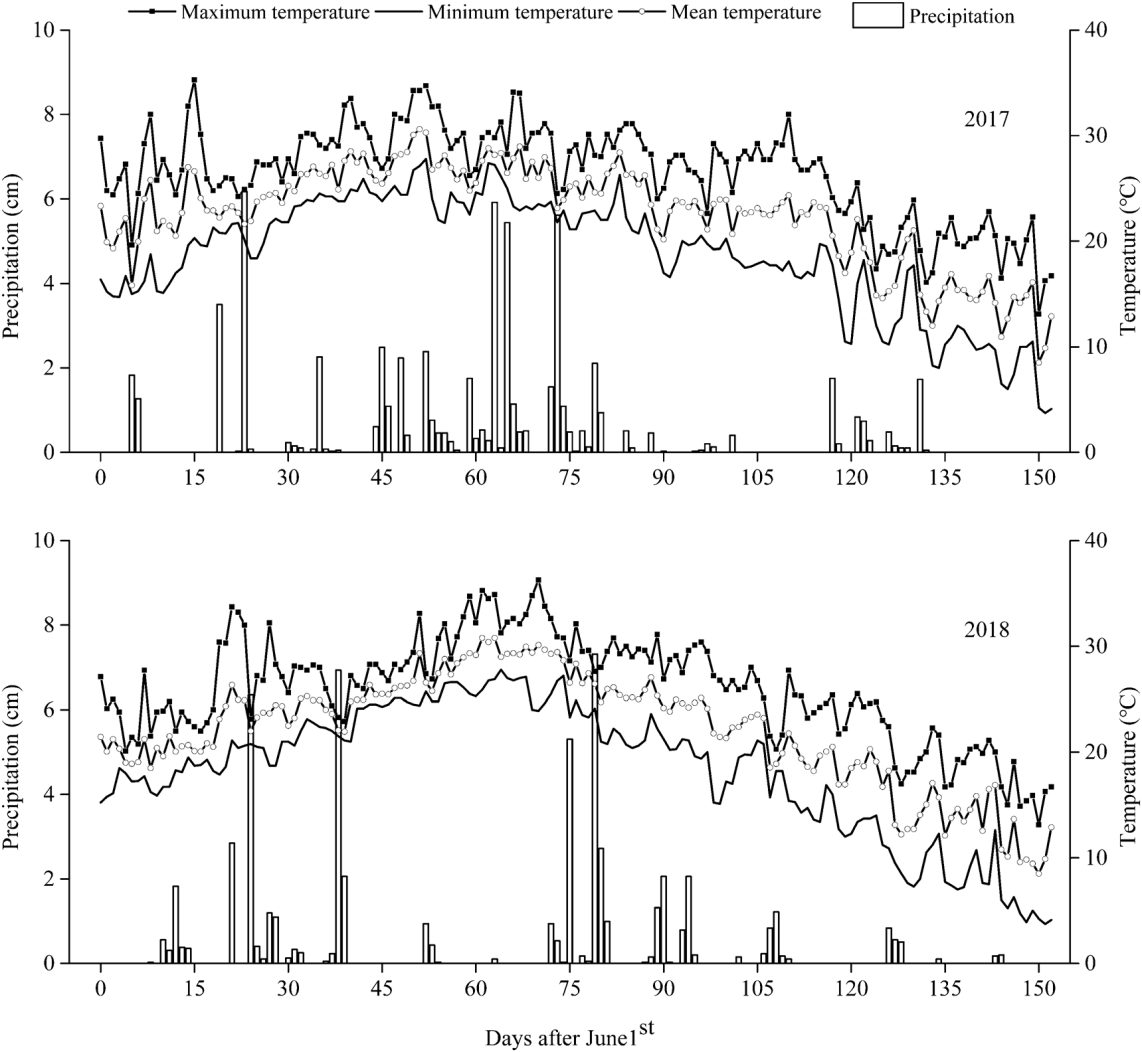
Precipitation (cm), maximum temperature, minimum temperature, mean temperature (℃) recorded during the growing seasons (from June 1st to October 30th) in 2017 and 2018.

Field experiments were conducted in 2017 and 2018, in Haiyang County (36° 78′ N, 121°16′ E), Shandong Province, China. The soil was classified as Fluvo-aquic soil. The site was characterized by fine-textured clay loam and well drained. The previously grown crop was wheat in 2017 and 2018. The nutrient status in the top 0–20 cm arable soil layer before seeding consisted of 21.84 g kg^−1^ organic matter (Walkley and Black method), 1.08 g kg^−1^ total N (Kjeldahl method), 41.74 mg kg^−1^ available P (Olsen method), 111.64 mg kg^−1^ available K (Dirks-Scheffer method). Soil pH was 5.44 as determined by acidity meter. The contents of sand, silt and clay were 470, 290, 240 g kg^−1^, respectively. Particle-size was analyzed using the hydrometer method after organic matter oxidation^29^. Precipitation and air temperature were measured by an automatic weather station (Fig. 3).

### Experimental design and field management

The field experimental treatments were arranged in a split plot design with three replications. According to our survey and the present study^15,30,31^, traditional nitrogen rate and planting density in smallholder fields in Huanghuaihai region in China were about 240 kg ha^−1^ and 60,000 plants ha^−1^, respectively. Based on these, the main plots were assigned to three N application rates (N1: 120 kg ha^−1^; N2: 180 kg ha^−1^ and N3: 240 kg ha^−1^), and subplots were designed to three planting densities (D1: 60,000 plants ha^−1^; D2: 75,000 plants ha^−1^ and D3: 90,000 plants ha^−1^). Every plot comprised of ten rows, 0.6 m spacing between rows and 8 m long. The plants in the rows were spaced at 0.278, 0.222, and 0.185 m, corresponding to 60,000, 75,000, and 90,000 plants ha^−1^, respectively. For the three N treatments, one half of the assigned amount, 60, 90, and 120 kg ha^−1^ (urea, N 46%) was applied as a basal fertilizer before sowing and the other half was applied at jointing stage. For all treatments, 80 kg phosphate (superphosphate, P_2_O_5_ 12%) and 160 kg potassium (potassium sulfate, K_2_O 50%) fertilizers were applied as a basal fertilizer.

Zhengdan 958, a widely released maize hybrid in Huanghuaihai region in China, was used. Zhengdan 958 was the offspring of inbred Zheng 58 and Chang 7-2 (deposition number 20000009), which are approved in China. In this study, the seeds of Zhengdan 958 were provided by Beijing Denong Seed Technology Co. Ltd. Experimental research and field studies on plants complied with relevant institutional, national, and international guidelines and legislation. Seeds were sown by hand, width two seeds per hole, on 22 June 2017 and 30 June 2018, respectively. The maize plants were thinned to one plant per hole to keep the designed densities one week after emergence. Crop management was the same as the local maize field. Maize was harvested on 9 October 2017 and 15 October 2018.

### Sampling and measurements

The dates on which about 75% plants were at tasseling stage and silking stage were recorded. Physiological maturity was judged by the appearance of seed black layer. Days to tasseling, silking and physiological maturity were counted as the days from emergence date to tasseling date, silking date and physiological maturity date, respectively.

At 12-leaf, silking and physiological maturity, five whole plants were sampled to dry to a constant weight and determine dry matter. At physiological maturity, maize ears were collected from the middle two rows per plot to measure grain number per ear and 1000-grain weight. Finally, maize ears were harvested from central area of 14.4 m^2^ per plot to measure ear number and grain yield. Profit was estimated using the following equation according to the method of Han et al.^20^.

1 $${\text{Profit }}\left( {\$ {\text{ ha}}^{{ - {1}}} } \right) \, = {\text{ Grain yield }} \times {\text{ grain price }} - {\text{ N rate }} \times {\text{ urea price }} - {\text{ seed rate }} \times {\text{ seed price}},$$

where the average price of maize grain, urea N, and maize seed in 2017 and 2018 was 269 $ t^−1^, 0.651 $ (kg urea-N)^−1^, and 1.5 $ (1000-seed)^−1^, respectively.

The whole plant of physiological maturity was divided to stalks, leaves, cob, and grain. The samples were dried, weighed, ground and digested with H_2_SO_4_–H_2_O_2_. N concentration was measured with the semi-micro Kjedahl method^32^. N uptake was estimated by multiplying the N concentration by the dry weight. PFP_N_ and NER were calculated as follows.

2 $${\text{PFP}}_{{\text{N}}} \left( {{\text{kg kg}}^{{ - {1}}} } \right) \, = {\text{ Grain yield}}/{\text{N rate}},$$

3 $${\text{NER }}\left( {{\text{kg kg}}^{{ - {1}}} } \right) \, = {\text{ Grain yield}}/{\text{N uptake}}.$$

### Statistical analysis

The data were statistically analyzed using DPS 7.05. Analysis of variance was conducted to evaluate the effects of N rate and planting density on the response variables. Means were compared using LSD test and differences were regarded as significance at *P* < 0.05. The trend surface simulation was analyzed using SPSS 19.0. Graphs were plotted using Matlab R2018a and OriginPro 9.0.
